# Remote Homology Detection Identifies a Eukaryotic RPA DBD-C-like DNA Binding Domain as a Conserved Feature of Archaeal Rpa1-Like Proteins

**DOI:** 10.3389/fmolb.2021.675229

**Published:** 2021-07-20

**Authors:** Stuart A. MacNeill

**Affiliations:** Biomedical Sciences Research Complex, School of Biology, University of St Andrews, St Andrews, United Kingdom

**Keywords:** RPA, OB fold, winged helix domain, archaea, ssDNA, DNA repair, DNA replication

## Abstract

The eukaryotic single-stranded DNA binding factor replication protein A (RPA) is essential for DNA replication, repair and recombination. RPA is a heterotrimer containing six related OB folds and a winged helix-turn-helix (wH) domain. The OB folds are designated DBD-A through DBD-F, with DBD-A through DBD-D being directly involved in ssDNA binding. DBD-C is located at the C-terminus of the RPA1 protein and has a distinctive structure that includes an integral C4 zinc finger, while the wH domain is found at the C-terminus of the RPA2 protein. Previously characterised archaeal RPA proteins fall into a number of classes with varying numbers of OB folds, but one widespread class includes proteins that contain a C4 or C3H zinc finger followed by a 100–120 amino acid C-terminal region reported to lack detectable sequence or structural similarity. Here, the sequences spanning this zinc finger and including the C-terminal region are shown to comprise a previously unrecognised DBD-C-like OB fold, confirming the evolutionary relatedness of this group of archaeal RPA proteins to eukaryotic RPA1. The evolutionary relationship between eukaryotic and archaeal RPA is further underscored by the presence of RPA2-like proteins comprising an OB fold and C-terminal winged helix (wH) domain in multiple species and crucially, suggests that several biochemically characterised archaeal RPA proteins previously thought to exist as monomers are likely to be RPA1-RPA2 heterodimers.

## Introduction

In all forms of life, single-stranded DNA binding proteins (SSBs) perform essential functions in DNA replication, repair and recombination by binding single-stranded DNA (ssDNA) with high affinity in a sequence-independent manner (Wold, 1997; Prakash and Borgstahl, 2012). SSB proteins are characterised by the presence of one or more structurally conserved OB (oligosaccharide-oligonucleotide-binding) fold domains. OB folds span 75–150 amino acids and consist of a five-stranded *ß*-sheet that is coiled to form a closed *ß*-barrel structure, often capped with an *α*-helix, with the heterogeneity in length reflecting the presence of variable regions generally located between the *ß*-strands (Wold, 1997; Prakash and Borgstahl, 2012).

**FIGURE 1:**
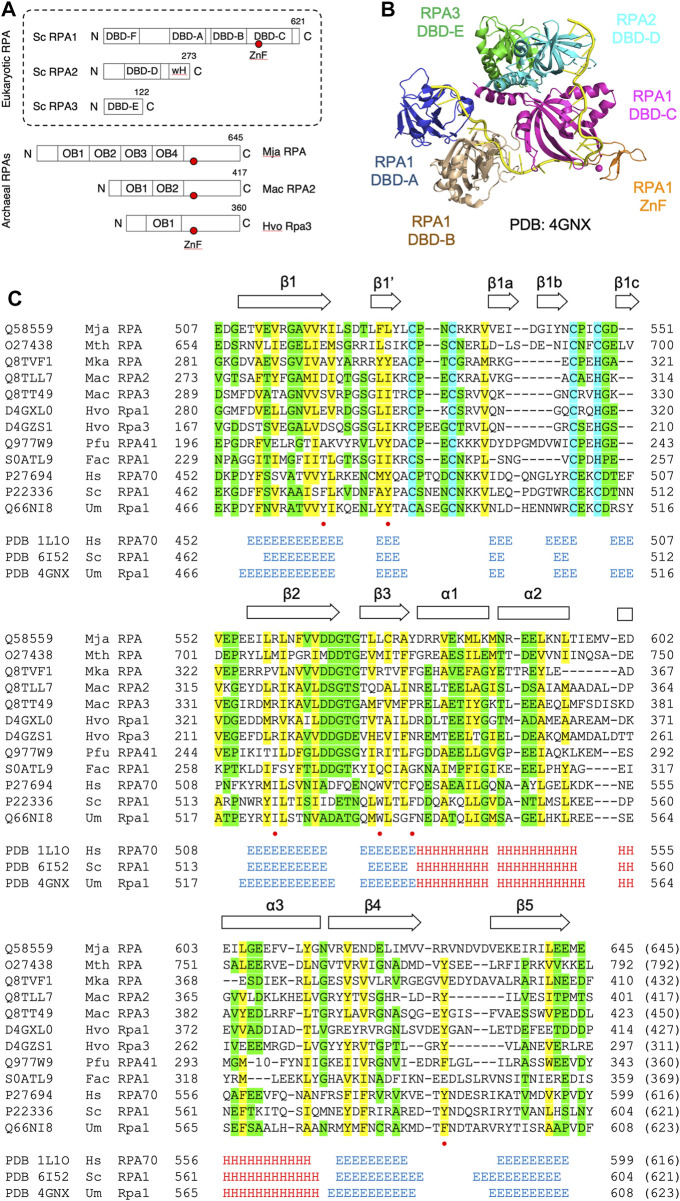
DBD-C-like OB fold in the archaeal RPA1-like proteins **(A)** Domain structure of eukaryotic and archaeal RPA proteins: *S. cerevisiae* RPA complex components RPA1, RPA2, and RPA3 (boxed with OB folds labelled DBD-A through-F) and representative archaeal zinc finger-containing RPA proteins from *Methanocaldococcus jannaschii* (MjaRPA), *Methanosarcina acetivorans* (MacRPA2), and *Haloferax volcanii* (HvoRpa3). The location of zinc finger (ZnF) and RPA2 winged helix (wH) domains are indicated **(B)** Crystal structure of the trimerization core of *Ustilago maydis* RPA bound to single-stranded DNA (PDB 4GNX) with OB folds from RPA1 (DBD-A, DBD-B, and DBD-C), RPA2 (DBD-D), and RPA3 (DBD-E) shown in different colours (Fan and Pavletich, 2012). The RPA1 zinc finger motif is shown in orange and single-stranded DNA in yellow **(C)** Protein sequence alignment of the C-terminal domains of archaeal and eukaryotic RPA proteins. UniProtKB or PDB accession numbers are shown on the left, followed by the organism and protein name. The positions of the first and last residues of the aligned region of the corresponding protein are indicated, with the number in brackets indicating the full length of the protein chain. Hydrophobic amino acids (AILVFMWYCH) are highlighted in yellow and smaller amino acids (GVACDENPST) highlighted in green when present in 9 of the 12 sequences shown. Residues (Y479, Y487, I524, W537, F541, F590) identified as being involved in ssDNA binding by the *U. maydis* RPA1 protein are indicated by red dots. The secondary structures of human, *S. cerevisiae* and *U. maydis* proteins are indicated (*H* = *α*-helix; E = *ß*-strand) and were taken from the indicated PDB entries. Abbreviations: Mja, *M. jannaschii*; Mth, *Methanothermobacter thermautotrophicus*; Mka, *Methanopyrus kandleri*; Mac, *M. acetivorans*; Hvo, *H. volcanii*; Pfu, *Pyrococcus furiosus*; Fac, *Ferroplasma acidarmanus*; Hs, human; Sc, *Saccharomyces cerevisiae*; and Um, *Ustilago maydis*.

In eukaryotes, the major single-stranded DNA binding factor is replication protein A (RPA) (Prakash and Borgstahl, 2012). RPA is a heterotrimeric complex made up of the RPA1, RPA2, and RPA3 proteins (also known RPA70, RPA32, and RPA14, respectively). RPA1 contains four OB fold domains (designated DBD-A, DBD-B, DBD-C, and DBD-F, where DBD indicates DNA binding domain), RPA2 contains one (DBD-D) and RPA3 one also (DBD-E) (shown schematically in Figure 1A) (Prakash and Borgstahl, 2012). DNA binding domains DBD-A, DBD-B, DBD-C, and DBD-D are primarily responsible for ssDNA binding by RPA, while DBD-E plays a structural role at the heart of the trimeric RPA complex and DBD-F functions as a site of protein-protein interaction (Figure 1B). RPA2 also possesses an extended N-terminal domain that is the target for regulatory phosphorylation and a C-terminal winged helix-turn-helix (wH) domain, both of which are also involved in protein-protein interactions. All three eukaryotic subunits have an *α*-helix at their C-terminal ends that together form a three-helix bundle that is essential for RPA trimer formation (Bochkareva et al., 2002).

The structure of DBD-C is markedly different from the other five DBDs. The DNA binding groove of DBD-C is wider and longer than that of DBD-A and DBD-B (Fan and Pavletich, 2012) and the amino acid sequence of the DBD-C OB fold is interrupted between *ß*-strands *β*1 and *β*2 by a zinc finger formed by three short antiparallel *ß*-strands (*β*1a, *β*2a, and *β*3a in Figure 1C) (Bochkareva et al., 2002; Fan and Pavletich, 2012). Residues from the zinc finger interact with the DNA phosphodiester backbone and may stabilise initial ssDNA binding by DBD-C (Fan and Pavletich, 2012). Loss of bound zinc causes destabilisation of RPA and a concomitant reduction in ssDNA binding (Bochkareva et al., 2000).

The archaea make up the third domain of life on Earth, are ubiquitous in nature and play important roles in biosphere and atmosphere (Jarrell et al., 2011). The evolutionary kinship of archaea and eukaryotes is undisputed and is particularly apparent in proteins involved in information storage and processing (DNA replication and repair, transcription and translation). Most archaeal lineages encode an RPA-like protein containing either a single OB fold or multiple OB fold domains in tandem, followed by a C4 or C3H zinc finger (ZnF) domain and a lengthy (100–120 amino acid) C-terminal region that has not previously been assigned a structure or function (Makarova and Koonin, 2013; Taib et al., 2021). Examples of biochemically characterised archaeal zinc finger-containing RPA proteins include *Methanocaldococcus jannaschii* RPA (MjaRPA) with four OB folds before the ZnF domain (Kelly et al., 1998), *Methanosarcina acetivorans* RPA2 (MacRPA2) with two OB folds before the ZnF (Robbins et al., 2004; Lin et al., 2005) and *Haloferax volcanii* Rpa3 (HvoRpa3) with a single OB fold before the zinc finger (Skowyra and MacNeill, 2012; Stroud et al., 2012) (diagrammed in Figure 1A). Evidence from *H. volcanii* indicates that the zinc finger-containing RPA proteins HvoRpa1 and HvoRpa3 share an essential function in the cell, despite the presence in this lineage of an essential non-zinc finger-containing RPA protein HvoRpa2 (Skowyra and MacNeill, 2012; Stroud et al., 2012). These proteins (HvoRpa1 and HvoRpa3) form heterodimeric complexes with their single OB fold-containing partner proteins HvoRpap1 and HvoRpap3, respectively (Stroud et al., 2012), while the *Pyrococcus furiosus* RPA41 protein forms a heterotrimeric RPA with RPA32 and RPA14 (Komori and Ishino, 2001). Other archaeal RPAs may exist as monomers or homomultimers.

Given the structural conservation of the OB folds in the N-terminal region of the archaeal zinc finger-containing RPA proteins, it is perhaps surprising that the C-terminal regions of these proteins have been reported to lack sequence similarity to eukaryotic RPA (Kelly et al., 1998; Kelman et al., 1999; Komori and Ishino, 2001; Robbins et al., 2004; Lin et al., 2005; Robbins et al., 2005; Lin et al., 2006; Lin et al., 2008; Pugh et al., 2008; Skowyra and MacNeill, 2012; Stroud et al., 2012). Here, the state-of-the-art protein sequence comparison and structure modelling tools HHpred (Zimmermann et al., 2017; Gabler et al., 2020) and MODELLER (Webb and Sali 2021) are used to re-analyse archaeal RPA protein sequences, allowing the extended C-terminal ZnF-containing region to be unambiguously identified as a DBD-C-like OB fold. This new analysis confirms the evolutionary relationship between multiple previously characterised archaeal RPA proteins and eukaryotic RPA1. Additional analysis confirming the presence of OB fold and wH domain-containing RPA2-like proteins encoded by linked or unlinked genes in multiple species highlights the need for further analysis of the archaeal RPAs that takes into account their likely heteromeric nature.

## Bioinformatic Analysis of Archaeal RPA Proteins

### The Archaeal RPA Zinc Finger Motif is Part of a DBD-C-Like OB Fold

Numerous archaeal RPA homologues have been identified that contain a C-terminal C4 or C3H ZnF motif followed by a 100–120 amino acid region with no known sequence similarity (Kelly et al., 1998; Kelman et al., 1999; Komori and Ishino, 2001; Robbins et al., 2004; Lin et al., 2005; Robbins et al., 2005; Lin et al., 2006; Lin et al., 2008; Pugh et al., 2008; Skowyra and MacNeill, 2012; Stroud et al., 2012). To re-investigate the nature of this region, HHpred (Zimmermann et al., 2017; Gabler et al., 2020) was used to search the PDB database, initially with the full-length 645 amino acid *M. jannaschii* RPA protein as the query and default search parameters. HHpred uses pairwise comparison of profile hidden Markov models (HMMs) for remote protein homology detection (Soding et al., 2005; Hildebrand et al., 2009). For MjaRPA, the top hit in this search was the large subunit of RPA from the corn smut fungus *Ustilago maydis*, Rpa1 (Table 1) (Fan and Pavletich, 2012). Significantly, the detected similarity extends well beyond the four previously recognised N-terminal OB folds (OB1–OB4) and the C4 zinc finger (Figure 1A) to include the C-terminal region of MjaRPA that had not previously been reported to display similarity to eukaryotic RPA proteins: amino acids 507–645 of MjaRPA are revealed to have similarity spanning DBD-C in *U. maydis* RPA1 (Fan and Pavletich, 2012), suggesting that the C-terminal region of MjaRPA constitutes a highly diverged DBD-C DNA binding domain. Similar HHpred searches with other previously characterised archaea zinc finger-containing RPA proteins produced similar results in all cases: a highly divergent DBD-C OB fold domain was identified where previously none had been detected, consistent with these proteins sharing a common DBD-C-containing ancestor (Table 1).

**TABLE 1:** Archaeal Rpa1-like protein similarity scores.

Protein	UniProt	Similarity to Um Rpa1 (PDB: 4GNX)	Similarity to Tt Teb1 (PDB: 7LMB)
Mja RPA	Q58559	2.9e−31	1.2e−29
Mth RPA	O27438	1.5e−30	3.7e−29
Mka RPA	Q8TVF1	4.0e−32	6.4e−31
Mac RPA2	Q8TLL7	1.3e−31	1.5e−31
Mac RPA3	Q8TT49	9.0e−34	1.5e−32
Hvo Rpa1	D4GXL0	3.4e−36	1.7e−35
Hvo Rpa3	D4GZS1	7.9e−22	3.1e−21
Pfu RPA41	Q977W9	3.8e−26	3.8e−25
Fac RPA1	S0ATL9	4.5e−36	8.7e−36

Searches were performed against the PDB_mmCIF70 database using HHpred with default search parameters. Scores are presented as E-values and reflect similarity across the full-length of the query proteins.

**FIGURE 2:**
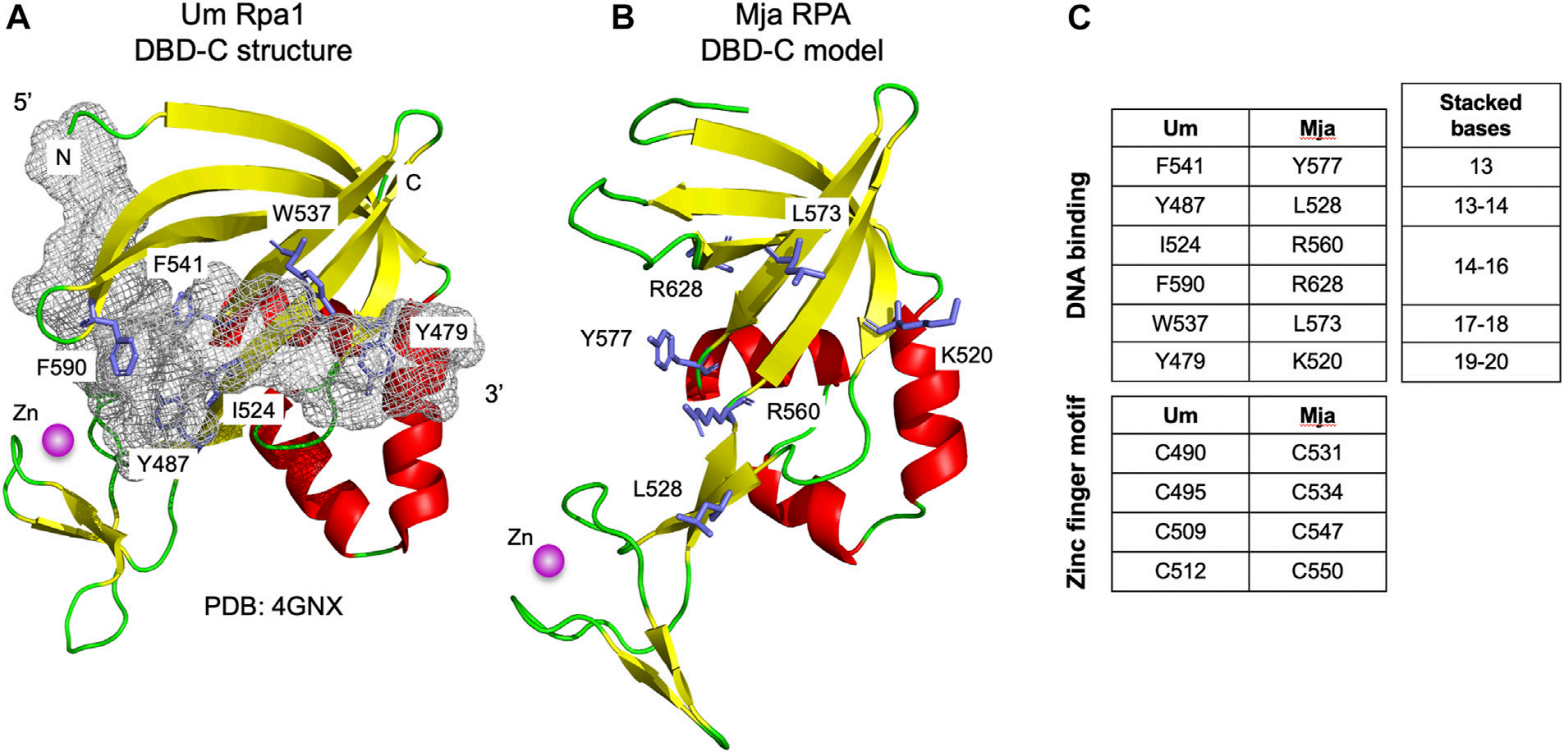
Modelled structure of Mja RPA DBD-C fold **(A)** Structure of *U. maydis* Rpa1 DBD-C fold bound to 16 nucleotides of ssDNA (shown as grey mesh, for clarity only bound nucleotides 10–25 from PDB: 4GNX are shown). Sidechains of amino acid residues implicated in DNA binding by base stacking are shown in stick form and labelled (see also Figure 1C). The zinc ion is shown is magenta **(B)** Modelled structure of *M. jannaschii* RPA DBD-C fold. The model was generated using HHpred (Zimmermann et al., 2017) and MODELLER 10.1 (Webb and Sali, 2021). Residues corresponding to the DNA binding residues in the *U. maydis* Rpa1 DBD-C are highlighted. **(C)** Conservation of ssDNA- and zinc-binding amino acids between Um Rpa1 and Mja RPA. The bases stacked by the amino acids listed are shown to the right.

Figure 1C presents a multiple sequence alignment of this region in previously characterised archaeal zinc finger-containing RPA proteins with eukaryotic RPA1 (RPA70). In each case the detected similarity supports the presence of all five *ß*-strands characteristic of the DBD-C OB fold (*β*1–*β*5), the short *ß*-strand *β*1,’ the three *α*-helices (*α*1–*α*3) located between *β*3 and *β*4, and the three short *ß*-strands (*β*1a–*β*1c) that form the zinc finger. Figure 2B shows the predicted structure for the *M. jannaschii* RPA DBD-C-like domain built using HHpred (Zimmermann et al., 2017; Gabler et al., 2020) and MODELLER (Webb and Sali, 2021). The sidechains of six hydrophobic amino acids in *U. maydis* RPA1 (Y479, Y487, I524, W537, F541, F590–see Figures 1C, 2A) have been identified as being involved in DBD-C ssDNA binding by base stacking. Some but not all of these are conserved in the archaeal RPA DBD-C domains, possibly reflecting significant differences in the mode of DNA binding used by the different proteins. In the *M. jannaschii* RPA DBD-C domain, for example, three of six hydrophobic sidechains (equivalent to F541, Y487, W537 in *U. maydis* Rpa1) are conserved, while the other three (I524, F590, and Y479) are replaced with basic sidechains. Whether these basic sidechains are involved in DNA binding remains to be seen.

### Winged Helix-Turn-Helix (wH) Domains are Found in RPA-Associated Proteins

**FIGURE 3:**
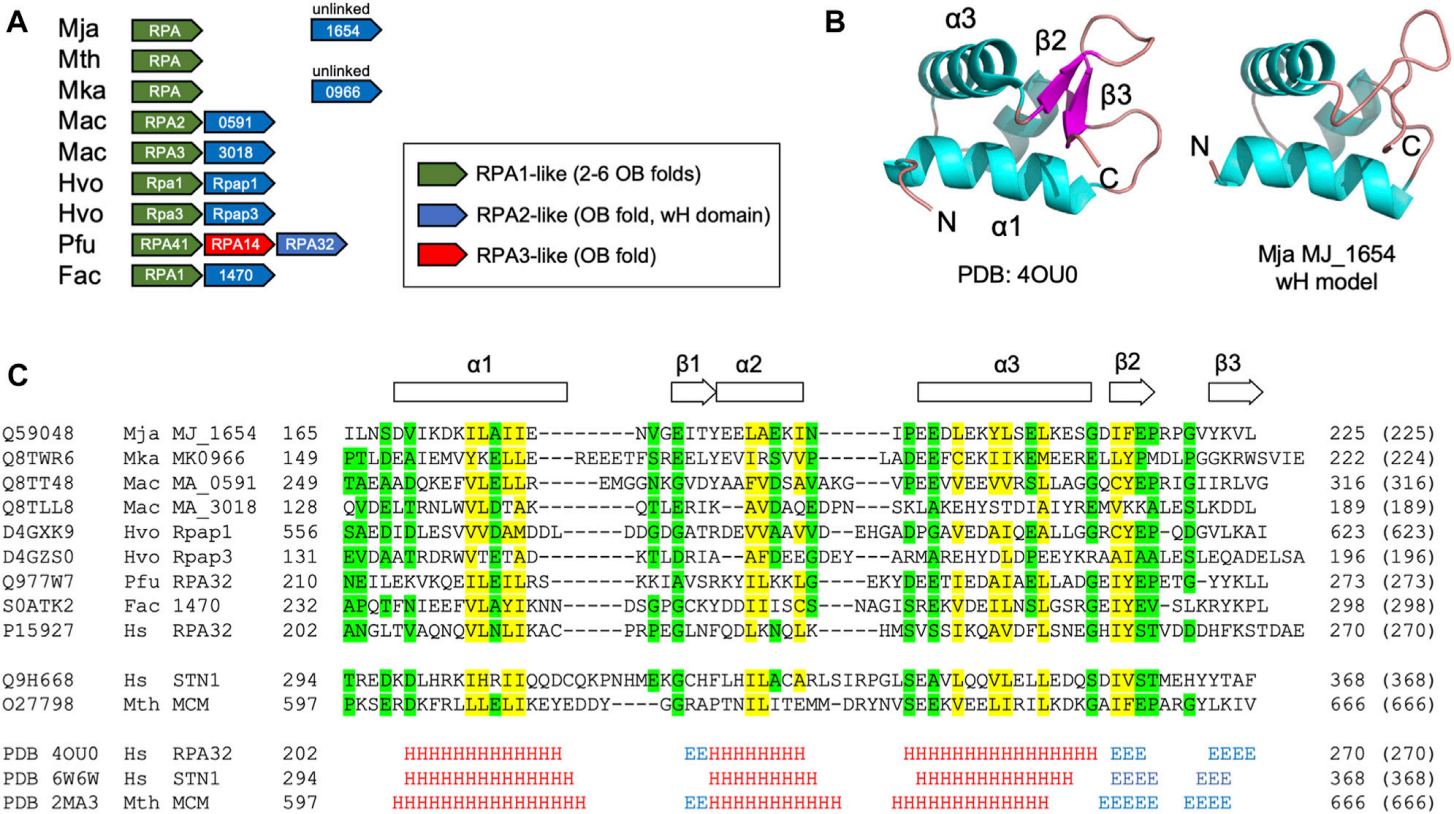
Winged helix-turn-helix (wH) domain in the archaeal RPA2-like proteins **(A)** Chromosomal organisation of ORFs encoding biochemically characterised archaeal RPA1-like and RPA2-like proteins (shown in green and blue, respectively). Linked (adjacent) and unlinked ORFs are shown **(B)** Right: Crystal structure of the wH domain at the C-terminus of the human RPA32 protein (PDB: 4OU0, RPA32 is the human orthologue of yeast RPA2). The structure comprises a three-helix bundle (*α*1–*α*3) capped by a *ß*-hairpin (*β*1–*β*3) (Feldkamp et al., 2014). Left: Structural model of the C-terminal wH domain (amino acids 169–225) of the *M. jannaschii* RPA2-like protein MJ_1654 based on its similarity to human RPA32 (amino acids 206–270, PDB: 4OU0). The model was generated using HHpred (Zimmermann et al., 2017) and MODELLER 10.1 (Webb and Sali, 2021) **(C)** Protein sequence alignment (as Figure 1, but with colouring applied when similar amino acids are present in seven of nine RPA2-like RPA proteins shown) of the putative wH domains of archaeal and eukaryotic RPA proteins, along with the human RPA2-like protein STN1 (Lim et al., 2020) and the *M. thermautotrophicus* MCM wH domains (Wiedemann et al., 2015). Alignments reflect the output of HHpred searches (Table 1) except for the Mac MA_318 and Hvo Rpap3 proteins which were manually aligned. The full ID of the *Ferroplasma acidarmanus* ORF/protein labelled 1470 is FACI_IFERC00001G1470. Abbreviations: Mja, *M. jannaschii*; Mth, *Methanothermobacter thermautotrophicus*; Mka, *Methanopyrus kandleri*; Mac, *M. acetivorans*; Hvo, *H. volcanii*; Pfu, *Pyrococcus furiosus*; Fac, *Ferroplasma acidarmanus*; Hs, human; Sc, *Saccharomyces cerevisiae*; and Um, *Ustilago maydis*.

The *Hfx. volcanii* RPA proteins Rpa1 and Rpa3 both contain C-terminal DBD-C-like OB-folds (Figure 1). These proteins have each been shown to form a stable complex with a single OB fold-containing protein encoded by an adjacent gene: Rpap1 in the case of Rpa1, and Rpap3 in the case of Rpa3 (Figure 3A) (Stroud et al., 2012). The exact stoichiometry of the Rpa1-Rpap1 and Rpa3-Rpap3 complexes is unknown. Aside from the presence of the single OB fold in Rpap1 and Rpap3, no other sequence motifs were detected in these proteins at the time of their initial characterisation (Skowyra and MacNeill, 2012; Stroud et al., 2012) but subsequent bioinformatic analysis suggested the presence of a C-terminal winged helix (wH) domain in these haloarchaeal proteins as well as in related proteins in other archaeal clades (Makarova and Koonin 2013).

Searching the PDB database using HHpred with the 623 amino acid *Hfx. volcanii* Rpap1 protein sequence as the query confirms the presence of the single OB fold towards the N-terminal end of Rpap1 (amino acids 1–155) and a wH domain at the C-terminal end (residues 561–623) that is related to those found in the human RPA32 and STN1 proteins (Table 2). As noted above, *Hfx. volcanii rpap1* is located adjacent on the chromosome to *rpa1*, with the two genes presumably co-transcribed (Skowyra and MacNeill, 2012; Stroud et al., 2012). The same is true of the *rpa3* and *rpap3* genes. Further inspection of sequence databases identifies genes encoding Rpap1/Rpap3 homologues adjacent to those encoding biochemically characterised DBD-C-containing RPA proteins in two highly divergent archaeal species: *M. acetivorans* (a representative of the Methanosarcinales) and *Ferroplasma acidarmanus* (Thermoplasmatales) (Figure 3B) (Robbins et al., 2004; Lin et al., 2005; Robbins et al., 2005; Lin et al., 2006; Lin et al., 2008). Using the sequences of these proteins in HHpred searches uncovers the predicted presence of an OB fold and a C-terminal wH domain in each (Table 2; Figure 3A), indicating that these RPA-associated proteins are RPA2 orthologues also. Unlinked genes encoding RPA2-like proteins are also found in other species from which DBD-C-containing RPA proteins have previously been characterised in isolation (*M. jannaschii* and *Methanopyrus kandleri*) but not in a third species, *Methanothermobacter thermautotrophicus* (Figure 3A) (Kelly et al., 1998; Kelman et al., 1999; Robbins et al., 2005).

**TABLE 2:** Archaeal Rpa2-like protein similarity scores.

Protein	UniProt	Similarity to Hs RPA32 (PDB: 2PI2)	Similarity to Hs STN1 (PDB: 6W6W)
Mja MJ_1654	Q59048	6.3e−28	1.2e−20
Mka MK0966	Q8TWR6	2.2e−29	N/D
Mac MA_0591	Q8TT48	1.3e−27	2.1e−21
Hvo Rpap1	D4GXK9	(0.013)	(0.19)
Pfu RPA32	Q977W7	9.4e−29	8.7e−23
Fac 1470	S0ATK2	4.4e−26	N/D

Searches were performed against the PDB_mmCIF70 database using HHpred with default search parameters. Scores are presented as E-values and reflect similarity across the full-length of the query proteins with the exception of the E-values in brackets for Hvo Rpap1 which indicate similarity over the putative C-terminal wH domain only. N/D, similarity to the wH domain of the RPA32-like protein STN1 not detected by HHpred search.

Figure 3C shows a multiple sequence alignment of the C-terminal region wH domain of eight archaeal RPA2-like proteins, showing conservation of sequences predicted to form all three α-helices (*α*1, *α*2, and *α*3) and two of three anticipated *ß*-strands (*β*1 and *β*2). This structural conservation is reflected in the predicted structure (Figure 3B) of the putative C-terminal wH domain in the *M. jannaschii* RPA2-like protein MJ_1654 (modelled based on the wH domain of human RPA2). Confirmation of the presence of the third anticipated *ß*-strand (*β*3) will require structural analysis of an archaeal RPA2-like wH domain.

## Discussion

The evolutionary kinship between the eukaryotic and archaeal lineages is clear, particularly in the information storage and processing systems (DNA replication and repair, transcription, translation). The data presented here serves to underline the similarity between a key player in eukaryotic replication, repair and recombination, the single-stranded DNA binding factor RPA, and its archaeal relatives, and serves to highlight the need for further analysis of archaeal RPA proteins that takes into account the presence of the previously undetected DBD-C OB fold and the fact that RPA1-like proteins previously thought to be monomeric (or possible homomultimeric) are more likely to be heterodimeric with a eukaryotic RPA2-like subunit harbouring both an OB fold and a C-terminal wH domain (Figures 1–3).

In the absence of focused biochemical and genetic analysis, the extent to which the DBD-C and wH domains contribute to archaeal RPA function is unclear. In eukaryotes, DBD-A and DBD-B were originally designated as high affinity DBDs while DBD-C and DBD-D were regarded as lower affinity DBDs, but recent results (Pokhrel et al., 2019) suggest that the situation is more complicated than this, with all subunits binding ssDNA with similar (high) affinities but with differing dynamics. No such analysis has been performed with archaeal RPA1-like proteins. Similarly, six hydrophobic amino acid residues (Y479, Y487, I524, W537, F541, F590) in the *U. maydis* RPA1 protein that have been identified as being involved in ssDNA binding by DBD-C (indicated by red dots in Figure 1C and shown in stick form in Figure 2A) are conserved to differing extents in various archaeal RPAs but whether these are involved in DNA binding remains to be seen. Truncation of the MacRPA3 protein immediately after the second OB fold removes the DBD-C domain in its entirety and results in a protein (MacRPA3-∆C152, corresponding to amino acids 1–298) that no longer binds zinc, as expected, but which retains near-normal ssDNA binding activity in fluorescence polarisation anisotropy (FPA) and electrophoretic mobility shift assays (EMSA) (Robbins et al., 2005). Near-normal ssDNA binding in FPA is also seen with an MkaRPA protein truncated after its second OB fold (amino acids 1–290) (Robbins et al., 2005). In contrast, mutating individual zinc-coordinating amino acids (C313, C316, C325 and H328, see Figure 1C) results in reduced DNA binding but also in more widespread structural disruption (Robbins et al., 2005). All these studies of archaeal proteins were performed with RPA1-like proteins in isolation, however, and not with RPA1-RPA2 complexes. The role of the wH domain is also unclear. In eukaryotic RPA, this mediates protein-protein interactions, so this is perhaps likely to be the case in archaea also (Wold, 1997; Prakash and Borgstahl, 2012). However, other roles, potentially including direct ssDNA binding, cannot be ruled out.

In summary, the presence of a previously undetected RPA1-like DBD-C OB fold and the RPA2-like wH domains in archaeal RPA proteins underscores the evolutionary kinship between these proteins and their eukaryotic counterparts RPA1 and RPA2, and has the potential to add significantly to our understanding of how these proteins perform their cellular function. To date, the three-dimensional structures of several archaeal RPA OB folds have been determined in isolation (PDBs 3DM3, 2K50, 3E0E, 2K5V, 2KEN, 2K75) but all are N-terminal OB folds and none of the structures includes bound ssDNA. In the short-term, determining the structure of an archaeal DBD-C-like OB fold, ideally bound to ssDNA, would be of great value. In the longer term, the structure of a full-length heterodimeric archaeal RPA bound to ssDNA will be required to fully appreciate how the OB folds mediate ssDNA binding and whether the DBD-C and wH domains have any part to play in this.

## Data Availability

The datasets presented in this study can be found in online repositories. The names of the repository/repositories and accession number(s) can be found in the article/supplementary material.
